# An Upcoming Global Challenge: Efficient Recycling for End‐of‐Life Lithium‐Ion Batteries

**DOI:** 10.1002/gch2.202200184

**Published:** 2022-12-14

**Authors:** Yan Wang, Huayi Yin, Liang An

**Affiliations:** ^1^ Department of Mechanical & Materials Engineering Worcester Polytechnic Institute Worcester MA 01609 USA; ^2^ School of Resource and Environmental Sciences Wuhan University Wuhan 430072 China; ^3^ Department of Mechanical Engineering The Hong Kong Polytechnic University Hung Hom Kowloon Hong Kong SAR 999077 China

Lithium‐ion batteries (LIBs) have been regarded as one of the most promising power sources and energy storage devices for portable electronics, large‐scale grid storage systems, and electric vehicles (EVs).^[^ ^1^, ^2^ ^]^ Notably, the number of LIBs for EVs has increased recently to deal with the increasingly severe global energy crisis and climate change. The global LIB energy storage capacity could increase to more than 2500 GWh (over 12.7 million tons) by 2030, which is almost ten times as much as that in 2019 of 218 GWh (over 1.2 million tons).^[^ ^3^ ^]^ However, the service life of current LIBs is only 3–5 years, meaning that numerous solid electrical and electronic wastes would be discarded in the following years.^[^ ^4^, ^5^ ^]^ In addition, typical LIB plants generate 5–10% of scrap material during the manufacturing process. Hence, the global LIB recycling market size is expected to reach almost US$11 billion by 2027.^[^ ^6^ ^]^ Commercial end‐of‐life (EOL) LIBs generally contain graphite anodes, metal oxide cathodes, as well as hazardous electrolytes and flammable additives, which would consequently create significant environmental concerns due to toxic heavy metals and harmful gases when they are not adequately handled.^[^ ^7^, ^8^ ^]^ On the other hand, these EOL LIBs could be regarded as precious secondary resources of important raw materials for new LIBs production, from recovered graphite reused as anodes, organic solvents for electrolytes, high‐content of Li salts and Ni/Co metal compounds for new cathodes, and Al/Cu foils reused as current collectors. So far, various circularity practices have been developed and fully studied, including remanufacturing, repurposing, industrial hydro‐/pyro‐metallurgy recycling, and lab‐scale direct recycling, to minimize LIB waste and avoid environmental pollution. Even though considerable and valuable Li, Co, and Ni metal compounds have been recovered from EOL LIBs, there are still some challenges to truly realize a circular LIB supply chain.^[^ ^11^ ^]^ Facing the upcoming Tremendous spent LIBs retired of LIBs, more global concerns should be focused on the management of EOL LIBs. This special issue features two perspective papers, two review papers, and two research papers that focus on the reclamation and reuse of cathode materials, organic electrolyte recycling, perspectives for efficient LIB recycling, safety concerns for management of EOL LIBs, and recycling strategies for next‐generation LIBs, with the aim to further promote the development of related technologies.

For typical EOL LIBs, the cathode part containing valuable Li, Co, and Ni in lithium cobalt oxide (LiCoO_2_, LCO) and lithium nickel manganese cobalt oxide (LiNi_x_Co_y_Mn_1–x–y_O_2_, NCM) covers the largest weight ratio (≈27%) and provides 40% of the economic value, such that all the recycling methods are mainly focused on the recovery of cathode materials in EOL LIBs. In this special issue, a research paper contributed by Dominika Gastol, Emma Kendrick, and co‐workers (article number 2200046) focuses on two different recycling routes; “shredding” and “disassembly” processes for reclamation and reuse of cathode materials from EOL LIBs. These two reclamation processes are evaluated based on the purity of reclaimed material, the performance of the remanufactured cell, and the energy required for the complete process, which proved that both disassembly and shredding routes are beneficial for recycling of EOL LIBs. Shredding can deal with any cell, including damaged, scrap, and EOL ones. However, the purity of waste streams is low in comparison to that from a disassembly process, while the disassembly process can only be applied to undamaged cells.

Apart from cathode materials, other components, such as graphite, binders, separators, organic electrolytes, and additives, are always burnt or abandoned in slag, which leads to huge emission of greenhouse gases and dust. In particular, the organic electrolytes in EOL LIBs easily react with air and water, causing severe secondary pollution and health threats toward humans. The organic electrolytes contain three parts: carbonate solvents, lithium salts, and additives, enabling the electrolyte to exhibit volatile, inflammable, toxic and sensitive properties. Generally, the aged electrolytes in EOL LIBs were diffused and penetrated into the electrode structure during continuous cycling, which are difficult to efficiently extract and collect.

In a review paper (article number 2200050), Liang An and co‐workers systematically summarize the recycling methods for aged electrolytes from EOL LIBs, including solvent extraction, supercritical and liquid CO_2_ extraction. Furthermore, the industrial developments are also compared, such as AEA Technology Batteries, OnTo Technology and Accurec. Based on these achievements, the authors present future perspectives toward aged electrolyte recycling, including potential electrolyte candidates, structure design for LIB electrodes, improvement for current recycling technologies, and establishment of a reliable and consistent business model from producers to consumers and finally to recyclers.

Although much progress has been achieved for cathode and electrolyte recycling in EOL LIBs, the global LIB recycling effort is still hampered by various factors such as insufficient logistics, regulation and technology readiness. A perspective paper contributed by Zheng Chen and co‐workers (article number 2200099) systematically summarizes the challenges associated with LIB recycling and their possible solutions from technical, political, economic, and environmental perspectives. Some promising strategies are presented to address these challenges and accelerate the emerging LIB recycling industry development.

Guangmin Zhou, Zheng Liang, and co‐workers also contributed a review paper (article number 2200067) to discuss the current progress, key issues and future prospects for LIB recycling. In this review paper, they first analyze the necessity for battery recycling from valuable resources in EOL LIBs, environmental issues, mineral mining, and uneven raw materials aspects. Secondly, the various LIBs recycling technologies that are currently used, such as pyrometallurgical and hydrometallurgical methods, are summarized and evaluated. In response to the challenges of above‐mentioned recycling methods, the authors look further into some of the cutting‐edge recycling technologies, such as direct repair and regeneration. In addition, the authors also discuss the prospects of selected recycling strategies for next‐generation LIBs such as solid‐state Li‐metal batteries.

Xin Qu, Huayi Yin, and co‐workers (article number 202200053) systematically study the mechanism of the ammonium sulfate roasting approach that can convent spent LIB cathode materials to water‐soluble salts at a relatively low temperature. Using the thermogravimetric analysis method, the authors obtained the reaction activation energies and roasting models. According to the results, the sulfate roasting process contains two steps: the phase boundary reaction dominates the first step and the nucleation reaction is the second step. The mechanistic understanding of ammonium sulfate roasting will shed light on developing efficient and green low‐temperature roasting approaches for LIB recycling.

Zhuowen Chen, Abdullah Yildizbasi, Yan Wang, and Joseph Sarkis (article number 2200049) seek to address the major safety oversight for EOL LIBs using closed‐loop supply chains that are critical to a larger circular economy environment, which focuses on the reverse flow within the closed‐loop supply chains, as this reverse flow is most closely aligned with EOL LIBs. The objective is to introduce and evaluate safety factors that affect LIB safety at each stage of the reverse‐flow portion of the supply chain. The work utilizes the technology‐organization‐environment (TOE) framework to help categorize safety factors in this area, which contributes to providing deep insights into LIB supply chains from the perspectives of circular economy and EOL management, raising awareness in scholarly research on safety concerns in LIB closed‐loop supply chains, and proposing potential research directions and a framework for investigations into safety problems with EOL LIB in the closed‐loop supply chain.

## Conflict of Interest

The authors declare no conflict of interest.
